# Nailfold Videocapillaroscopy in Connective Tissue Diseases with Raynaud’s Phenomenon in an Indian Population

**DOI:** 10.5041/RMMJ.10460

**Published:** 2022-01-27

**Authors:** Sambit Sundaray, Siddhartha Mishra, Subhash Chandra Dash, Naba Kishore Sundaray

**Affiliations:** 1 Department of Nephrology, All India Institute of Medical Sciences, New Delhi, India; 2 Department of Medicine, Kalinga Institute of Medical Sciences, Bhubaneswar, India; 3 Department of Medicine, Institute of Medical Sciences and SUM Hospital, Bhubaneswar, India

**Keywords:** Connective tissue diseases, nailfold capillaroscopy, Raynaud’s phenomenon, scleroderma pattern, systemic sclerosis, videocapillaroscope

## Abstract

**Introduction:**

Microvasculopathy is characterized by progressive structural and functional damage to the microvessels and plays a key role in the pathogenesis of various connective tissue diseases (CTD). Nailfold videocapillaroscopy is an optimal and validated method for analysis of microvascular abnormalities and is able to differentiate secondary Raynaud’s phenomenon (RP) of CTD from primary RP and healthy subjects.

**Aim:**

To assess and analyze nailfold capillaroscopic findings in Indian subjects with secondary Raynaud and to compare with findings in healthy subjects.

**Methods:**

A total of 62 study participants including cases and controls underwent nailfold videocapillaroscopy. Capillary loop length, capillary width, capillary density, presence/absence of tortuosity, giant loops, neoangiogenesis, microhemorrhages, and avascular areas were the parameters studied.

**Results:**

All the quantitative and qualitative parameters studied were significantly associated with secondary RP. Mean loop length in cases of connective tissue diseases was significantly less than in the controls (225.74 μm versus 282.97 μm) (*P*=0.002). Capillary density was also reduced significantly in the cases as compared to the controls (4.6 versus 7.39/mm) (*P*<0.01), whereas it was markedly decreased in systemic sclerosis (SSc) and mixed connective tissue diseases (MCTD), and near normal in systemic lupus erythematosus (SLE). Tortuosity was the most frequent (77.4%) qualitative parameter. Scleroderma pattern was found in 62.5% of patients with SSc and in 60% with MCTD. Non-specific pattern was found in 80% of SLE cases and 50% of dermatomyositis cases.

**Conclusion:**

Both quantitative and qualitative capillaroscopic changes are significantly associated with secondary RP. Scleroderma pattern was predominant in SSc and MCTD, whereas non-specific pattern was predominantly found in SLE and dermatomyositis.

## INTRODUCTION

Microvascular abnormalities are the hallmarks of various connective tissue diseases (CTD) that present with Raynaud’s phenomenon (RP). In systemic sclerosis (SSc), secondary RP is the initial or heralding symptom in over 90% of patients, and in systemic lupus erythematosus (SLE) in up to 45% of patients.^1^ Presence of pulmonary arterial hypertension indicates more pronounced (endothelial-dependent) microvascular dysfunction contributing to the increased mortality risk in SSc patients.^2^ Microvasculopathy plays a central role in the development of RP, digital ulcers, and pulmonary arterial hypertension, thus rendering the evaluation of the peripheral microvasculature obligatory for early diagnosis and follow-up in SSc patients.^1^,^3^

Nailfold videocapillaroscopy (NVC) is an optimal, validated, and reproducible method for qualitative and quantitative assessment of nailfold capillary abnormalities, thus differentiating secondary RP due to CTD from primary RP and healthy individuals.^3^–^5^ Secondary RP is characterized by abnormal capillaroscopic findings, reflecting microvascular damage. Typical capillaroscopic findings referred to as scleroderma pattern is observed predominantly in SSc, and with lesser frequency in other CTDs too.^6^ Moreover, NVC can identify morphologic patterns specific to different stages of SSc.^3^,^5^ Further, NVC screening at regular interval (6–12 months) is recommended for primary RP to get the early signs of transitioning to secondary RP.^3^,^7^ In primary RP, NVC pattern is frequently normal, but presence of one or more abnormal capillaroscopic findings such as progressive capillary dilation raises the possibility of transition to secondary RP.^5^ Besides, capillary density being an important parameter of NVC reduces in SSc in correlation with the severity of internal organ involvement, and it is not influenced by the presence of overlap syndrome.^6^ Capillaroscopy is also useful to predict the development of digit ulceration in SSc and to differentiate dermatomyositis from polymyositis.^7^,^8^ Nailfold videocapillaroscopy not only detects the disease early and predicts the future evolution but can be utilized to monitor the response to therapy as well.^3^,^9^

In recognition of its importance, abnormal capillaroscopy was included in the diagnostic criteria of SSc by the European League Against Rheumatism and the American College of Rheumatism in 2013.^10^ Since 2014, absence of typical nailfold capillary changes is a criterion in diagnosing primary RP, and the consensus on workup of patients with RP is to have a nailfold capillaroscopic examination to characterize the clinical profile.^11^

Though several studies are available in Western literature, studies in India are very few, and that too by videocapillaroscope is scant. The present study aimed to assess and analyze the nailfold capillary changes in connective tissue diseases with RP and compare results with those in healthy controls.

## MATERIAL AND METHODS

An observational case-control study was conducted in Armed Forces Medical College, Pune on 62 study participants after obtaining the due approval from the institutional ethics committee (Ref. no. IEC/2248/13). Consecutive cases of previously known CTDs with RP, aged above 18 years, attending the out-patient departments during January 2015 to January 2016, were included in the study. Patients with diabetes mellitus, ulcerated fingers, history of cosmetic surgery in nailfolds within 3 months, and smokers were excluded from the study. A total of 31 patients were enrolled for the study. For control, an equal number of age- and sex-matched healthy individuals with no evidence of CTD were selected. Both cases and controls had the details of the study explained to them, and written informed consents were obtained.

**Figure 1:**
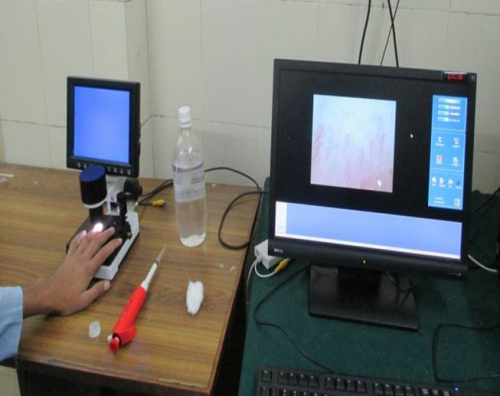
Nailfold Videocapillaroscopy.

Participants’ old medical documents were perused, followed by detailed history-taking and a thorough clinical examination to collect relevant data confirming the diagnoses. Participants were then subjected to nailfold capillaroscopy (NFC) after spending a minimum of 20 minutes in an enclosed room with a steady temperature of 20–23°C. They were instructed to refrain from caffeinated drinks 4 hours prior to the examination. Nailfold videocapillaroscopy was performed using Videocapillaroscope (JH-1004 C model, Jiangsu Jiahua Electronic Instrument Co., Ltd, Xuzhou, Jingasu, China) consisting of a microscope with ×380 magnification coupled to a digital video camera. Participants were asked to gently wash their hands with mild soap and water. Immersion oil was then applied to improve visualization of the capillaries. All fingers of both hands (except thumbs) were then placed on the microscope stage. Fingers affected by recent trauma were not analyzed. The ring finger was chosen for quantitative assessment because it tends to be less traumatized and has a 3–5 mm length of proximal nailfold. The nailfolds were scanned and analyzed using a computer-based analytical system with the aid of specific software bundled with the hardware (Figure 1).

The capillary morphology, arrangement, density, length, and width were assessed. The quantitative and qualitative parameters were carefully defined after the literature search.^4^,^5^,^12^–^15^ The mean capillary length was calculated as an average of the lengths of the three most clearly visible consecutive capillaries in the fourth finger of both hands. Capillary loop length of >500 μm was defined as loop elongation. The width of the arterial and venous limbs was taken in three of the largest appearing capillaries, and finally the mean width was calculated. Capillary with irregularly enlarged diameter of ≥20 μm (and <50 μm) was defined as dilated capillary, and homogeneously enlarged capillary with diameter of ≥50 μm as giant capillary loop. Mean capillary density was calculated as an average of readings (number of capillary loops per linear mm measured in the distal row) in the majority of the eight capillary beds assessed. Presence of avascular areas (areas where two or more consecutive capillaries are absent), microhemorrhages, tortuous, ramified, bushy, branched capillaries, and capillary disorganization (architectural disorientation) were assessed and analyzed. Tortuous or crossing capillaries and dilated capillaries were considered as minor morphological abnormalities. Major abnormalities included giant capillaries and ramified, branched (neoangiogenesis), and meandering capillaries. Normal capillary pattern was defined as homogeneous distribution of 7–12 hairpin-shaped capillaries per linear mm or with minor morphologic abnormalities.^12^,^15^ The scleroderma patterns were defined as “early pattern” with few giant capillaries, few microhemorrhages, no avascular area, and normal capillary architecture; “active pattern” with numerous giant capillaries, frequent microhemorrhages, mildly disorganized capillary architecture, moderate capillary loss, and few ramified capillaries; and “late pattern” with the presence of severely disorganized capillaries, extensive avascular areas, frequent neoangiogenesis, nearly absent microhemorrhage, and giant capillaries. Presence of highly tortuous (meandering), crossed or dilated capillaries with variable length (non-homogeneous distribution) and microhemorrhages was defined as non-specific pattern.

### Statistical Analysis

Data analysis was done by using SPSS version 20.0. After normality test, categorical data were expressed with number and percentage. Chi-square and Fisher’s exact tests were used to compare the qualitative parameters between cases and controls. Continuous data were expressed with mean and standard deviation. Independent *t* test for comparison of quantitative data between the case and control groups and ANOVA test for more than two groups was used. Two-tailed *P* value of <0.05 was considered as significant.

## RESULTS

A total of 62 study participants underwent NVC. The mean age of cases was 41.48±15.8 years and was not different from controls statistically (41.81±12.9 years) (*P*=0.930). Females constituted 71% of cases and 77% of controls (*P*=0.562). The majority of patients with secondary Raynaud (51.6%, *n*=16) had SSc, out of which 37.5% (*n*=6) had diffuse cutaneous SSc (DcSSc) and 62.5% (*n*=10) had limited cutaneous SSc (LcSSc). Other characteristics are given in Table 1.

**Table 1:** Demographic Characteristics of the Study Participants (*n*=62).

Characteristics	Cases (*n*=31)	Controls (*n*=31)	*P* Value
Age (years), mean±SD	41.48±15.88	41.81±12.94	0.930*
Female Sex % (*n*)	71% (22)	77% (24)	0.562†
Disease duration (years)	5.77±4.84		
DcSSc % (*n*)	19.4% (6)		
LcSSc % (*n*)	32.3% (10)		
SLE% (*n*)	16.1% (5)		
MCTD % (*n*)	16.1% (5)		
DM % (*n*)	12.9% (4)		
UCTD % (*n*)	3.2% (1)		

* Independent *t* test.

† Chi-square test.

DcSSc, diffuse cutaneous systemic sclerosis; DM, dermatomyositis; LcSSc, limited cutaneous systemic sclerosis; MCTD, mixed connective tissue diseases; SD, standard deviation; UCTD, undifferentiated connective tissue diseases.

**Figure 2:**
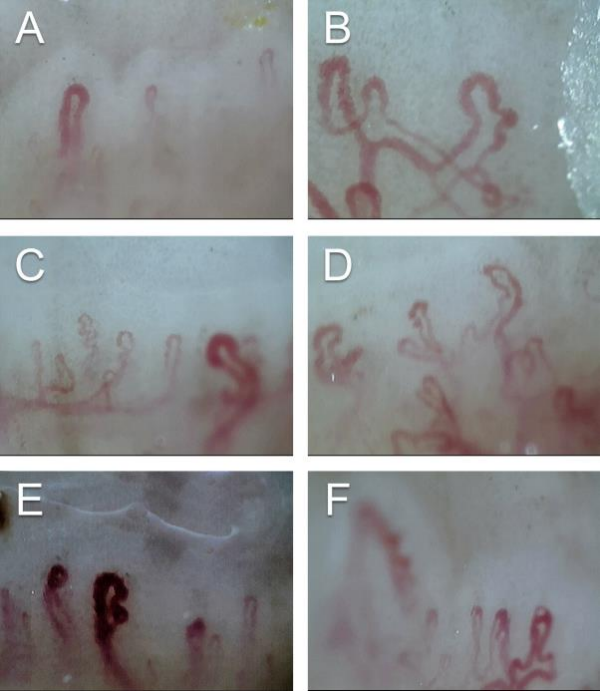
Capillaroscopic Findings of Morphologic Aberrations. **A:** Avascular areas; **B:** Neoangiogenesis; **C:** Dilated capillary; **D:** Ramified capillaries; **E:** Giant capillary; **F:** Tortuosity.

The results of NVC were as follows:

1. Mean capillary loop length in connective tissue diseases (CTDs) was significantly less than that of the controls (282.97±16.26 μm) (*P*=0.002). It was 248.1±77.5 μm in diffuse cutaneous sclerosis (DcSSc), 216.50±80.25 μm in limited cutaneous systemic sclerosis (LcSSc), 228.60±35.4 μm in SLE, 217.60±45.86 μm in mixed connective tissue diseases (MCTD), and 173 μm in undifferentiated connective tissue diseases (UCTD) (Table 2).
2. Mean loop width in the CTD patients was significantly more than that in controls (13.13 μm) (*P*<0.001). The diameter was widest in cases with LcSSc (40.5±21.7 μm). The mean loop widths of other CTD patients are given in Table 2.
3. Capillary density was also reduced significantly in the patient group as compared to controls (7.39/mm) (*P*<0.001). It was least in diffuse SSc (3.0±1.5/mm) followed by MCTD (3.2±2.28) (Table 2).
4. Tortuosity was observed in 77.4% of patients (*n*=24) and 10% of controls (*n*=3), which was significant (*P*<0.001) (Table 2).
5. Giant capillaries (Figure 2) were observed in 22.6% (*n*=7) of CTDs. It was found in 40% (*n*=4) of LcSSc cases, 16.6% (*n*=1) of DcSSc, 40% (*n*=2) of SLE, and nil in controls (*P*=0.004). Dilated capillaries were found in 60% (*n*=3) of MCTD, 40% (*n*=2) of SLE, 33.3% (*n*=2) of DcSSc, 40% (*n*=4) of LcSSc, and none in controls (*P*<0.001).
6. Microhemorrhages were observed in 40% (*n*=2) of SLE, 25% (*n*=1) of dermatomyositis, 20% (*n*= 2) cases of LcSSc, and nil in controls (*P*=0.005) (Table 2).
7. Neoangiogenesis (Figure 2) was noted in a total of 15 cases (48%). Disease-wise, it was more frequent in SSc (56.2%), dermatomyositis (50%), SLE (40%), and MCTD (40%), while none was noted in controls (*P*<0.001) (Table 2).
8. Avascular areas (Figure 2) were observed in 58% of cases (*n*=18) and none in controls (*P*<0.001). Patients with SSc (*n*=10) and MCTD (*n*=5) showed higher frequency of capillary loss (Table 2).
9. The scleroderma pattern was found in 62.5% of SSc and 60% of MCTD cases. Early pattern constituted 50% (*n*=7) of scleroderma pattern (Table 3). The non-specific pattern was found in 80% (*n*=4) of SLE cases (Table 2).

## DISCUSSION

In the present study, most of the patients were over 30 years of age and had had their disease for about 5 years. Characteristically, secondary RP usually occurs after the age of 30 years.^16^ The study population was predominantly female (71%), in keeping with the general trend of systemic connective tissue diseases being more prevalent in females. Nailfold videocapillaroscopy is an easier, safer, non-invasive, and most optimal method to study the capillary changes. All the quantitative and qualitative parameters studied were significantly associated with secondary RP. The capillary lengths were significantly reduced in patients compared with healthy controls and markedly reduced in UCTD, LcSSc, and MCTD (*P*=0.002). However, previous Indian studies had not commented on capillary loop length.^17^,^18^ Capillary density was also reduced significantly as compared to the control group (*P*<0.001). It was markedly decreased in SSc and MCTD, near normal in SLE, and near the lower limit of normal in UCTD.

**Table 2:** Nailfold Capillaroscopic Changes Among Cases and Controls.

Parameters	Controls (*n*=31)	DcSSc (*n*=6)	LcSSc (*n*=10)	SLE (*n*=5)	MCTD (*n*=5)	DM (*n*=4)	UCTD (*n*=1)	Cases (*n*=31)	*P* Value
Mean loop length (μm)	282.97± 28.94	248.17±77.57	216.50±80.25	228.60±35.47	217.60±45.86	235±25.83	173	225.74±60.79	0.002*
Mean loop width (μm)	13.13±3.44	25±19.92	40.50±21.79	34.20±16.63	19.20±7.53	19.50±12.12	12.0	29.42±18.90	0.0001*
Mean capillary density (/mm)	7.39±1.3	3.0±1.55	4.7±2.06	6.6±2.88	3.2±2.28	5.0±2.16	9.0	4.61±2.47	0.0001*
Tortuosity % (*n*)	10% (3)	100% (6)	80% (8)	80% (4)	60% (3)	50% (2)	100% (1)	77% (24)	0.0001†
Giant capillary % (*n*)	0% (0)	16.6% (1)	40% (4)	40% (2)	0% (0)	0% (0)	0% (0)	22.6% (7)	0.004†
Dilated capillary % (*n*)	0% (0)	33.3% (2)	40% (4)	40% (2)	60% (3)	25% (1)	0% (0)	38.7% (12)	0.0001†
Microhemorrhage % (*n*)	0% (0)	0% (0)	20% (2)	40% (2)	0% (0)	25% (1)	0% (0)	16% (5)	0.005†
Neoangiogenesis % (*n*)	0% (0)	66.6% (4)	50% (5)	40% (2)	40% (2)	50% (2)	0% (0)	48% (15)	0.0001†
Avascular area % (*n*)	0% (0)	83.3% (5)	50% (5)	20% (1)	100% (5)	50% (2)	0% (0)	58.0% (18)	0.0001†
Scleroderma pattern % (*n*)	0% (0)	66.6% (4)	60% (6)	0% (0)	60% (3)	25% (1)	0% (0)	45.1% (14)	0.0001†
Non-specific pattern % (*n*)	0% (0)	33.3% (2)	30% (3)	80% (4)	40% (2)	50% (2)	100% (1)	45.1% (14)	0.0001†

* ANOVA test.

† Fisher’s exact test.

DcSSc, diffuse cutaneous systemic sclerosis; DM, diabetes mellitus; LcSSc, limited cutaneous systemic sclerosis; MCTD, mixed connective tissue disease; SLE, systemic lupus erythematosus; UCTD, undifferentiated connective tissue disease.

**Table 3:** Scleroderma Patterns in Connective Tissue Diseases.

Scleroderma Patterns	Total (*n*=14)	DcSSc (*n*=6)	LcSSc (*n*=10)	SLE (*n*=5)	MCTD (*n*=5)	DM (*n*=4)	UCTD (*n*=1)
Early Pattern	50% (7)	33.3% (2)	30% (3)	0	20% (1)	25% (1)	0
Active Pattern	21.4% (3)	16.6% (1)	10% (1)	0	20% (1)	0	0
Late Pattern	28.6% (4)	33.3% (2)	10% (1)	0	20% (1)	0	0

DcSSc, diffuse cutaneous systemic sclerosis; DM, dermatomyositis; LcSSc, limited cutaneous systemic sclerosis; MCTD, mixed connective tissue diseases; SLE, systemic lupus erythematosus; UCTD, undifferentiated connective tissue diseases.

Systemic sclerosis constituted 51.6% of the cases in the present study. Autoimmunity, vasculopathy, and progressive fibrosis characterize SSc. Anti-endothelial cell antibody (AECA), anti-angiotensin II receptor, and anti-endothelin type-A receptor antibody are the autoantibodies involved in endothelial cell injury, which is probably the critical initial event in the pathogenesis of vasculopathy. Further, AECA is associated with a higher rate of mortality in SSc and can be found in up to 85% of cases.^1^ Microvasculopathy is more pronounced and plays a pivotal role in the development of pulmonary hypertension, vascular ectasia, coronary vascular disease, renal crisis, and digital ulcers. The degree of nailfold capillary changes can be a guide to early diagnosis, disease progression, and prognosis in SSc.^3^,^7^ The predominant NVC findings in our study were capillary loss, tortuous capillaries, and neoangiogenesis in SSc cases. Other Indian studies^17^,^18^ observed avascular areas slightly more frequently (81%–94% of DcSSc and 25%–41% of LcSSc patients), whereas a recent Asian study^19^ reported a lower frequency of avascular areas (46.7% of DcSSc and 36.4% of LcSSc) in SSc patients. Tortuosity was found in a higher percentage of SSc cases in our study than a previous study by Bhakuni et al.,^17^ and the frequency of neoangiogenesis is similar to that of a study by Shenavandeh et al.^19^ Among the various proangiogenic factors, vascular endothelial growth factor (VEGF) is typically involved in several processes of physiologic and pathologic angiogenesis and associated with decreased nailfold capillary density in SSc.^4^ Capillary density was found to be reduced significantly (3± 1.5/mm in DcSSc and 4.7±2/mm in LcSSc) in our study, which is in concordance with the observation by Jakhar et al. in their study.^18^ However, another Indian study^17^ demonstrated higher capillary density in their patients as compared to our observation. Giant capillaries and microhemorrhages were found less frequently as compared to the previous Indian studies.^17^,^18^ The possible explanation of variations in frequency of NVC parameters in various studies may be due to varying sample size, duration of RP, and use of different equipment with different magnification in the studies. Presence of more than one morphological sign of giant capillaries, microhemorrhages, avascular areas, and angiogenesis is suggestive of microvascular damage. These sequential capillaroscopic changes are typically observed in SSc patients and described as scleroderma pattern. The present study observed a scleroderma pattern in 83.3% of DcSSc and 50% of LcSSc cases, which is similar to the observation (81% of DcSSc and 75% of LcSSc) by Bhakuni et al.^17^ With the help of NVC (early pattern), SSc can be detected much before the onset of clinical manifestations. Further, active and late scleroderma patterns correspond to organ involvement.^7^ The early scleroderma pattern was found in 31.2%, with active and late pattern in 12.5% and 18.7%, respectively, in our study. In MCTD, capillary involvement varies widely, and NFC is a dynamic process.^7^,^20^ Nonetheless, the scleroderma pattern is predominant and is found in up to 60% cases.^21^ This study also observed the scleroderma pattern in 60% cases. Prominent NFC findings were capillary loss, tortuosity, dilated capillaries, and neoangiogenesis. The scleroderma pattern occurs occasionally in UCTD.^22^ The present study included only one UCTD case, and the NVC pattern was of the non-specific type.

Vascular involvement is typically in the form of both vasculopathy and vasculitis and is the leading cause of death in SLE. Anti-endothelial cell antibodies are found in 80% of cases and are considered to be involved in microvascular dysfunction.^1^ Ingegnoli reported that nailfold capillary changes are often normal, and subtle changes occur in about 30% cases.^23^ Conversely, a subsequent study by Ragab et al.^24^ reported that non-specific changes can occur in about 75% of SLE. The present study observed tortuous capillaries as the most frequent capillary changes, and the predominant NFC pattern was the non-specific pattern. Moreover, a recent study by Bernardino et al.^25^ reported that 79% of SLE patients had non-scleroderma pattern, with tortuosity (98%) and hemorrhages (52%) as the main NFC findings, which supports our study findings.

In dermatomyositis, bushy, ramified capillaries and neoangiogenesis are common, and scleroderma pattern occurs in 20%–60% of cases.^15^,^26^ However, the capillary changes can revert to normal in remission and in response to treatment, whereas persistence of NFC confers poor prognosis.^21^,^27^ Tortuous capillaries, neoangiogenesis, and avascular areas are the main findings in our study, whereas an Asian study^28^ reported microhemorrhages, avascular areas, and neoangiogenesis as the main findings and scleroderma pattern in 88.9% cases. In contrast, our study found non-specific pattern in 50% and scleroderma pattern in 25% of patients. However, Indian studies on NFC in dermatomyositis are scarce. Whether the NFC patterns and changes have racial and geographic variations is a matter for further studies.

A limitation of the present study is that this is a single-center study with a smaller sample size, raising the possibility of bias in selection of patients. Our study also did not evaluate for progression of capillary changes or treatment response. An advantage of this study is that it included rare CTDs and an equal number of healthy controls for comparison, thereby providing a valuable data source that could be useful for further studies. However, multi-center studies with a larger sample size are required for generalization of the findings of this study.

## CONCLUSION

Both quantitative and qualitative capillaroscopic changes are significantly associated with secondary Raynaud’s phenomenon. The nailfold videocapillaroscopy pattern in systemic sclerosis is predominantly a scleroderma pattern. Giant capillaries are dominant. Capillary loss is severe in diffuse type. Tortuosity and neoangiogenesis are frequent. Microhemorrhages are significantly less common. In mixed connective tissue diseases, scleroderma pattern is frequent. Giant capillaries are not noted, though capillary dilatation is frequent. Capillary loss is a frequent finding. In SLE, the findings are non-specific pattern. Tortuosity is most frequent. Capillary density is near normal. In dermatomyositis, capillary density is slightly reduced, and the nailfold videocapillaroscopy findings are non-specific patterns.

## Abbreviations

CTD: connective tissue disease
DcSSc: diffuse cutaneous systemic sclerosis
LcSSc: limited cutaneous systemic sclerosis
MCTD: mixed connective tissue disease
NFC: nailfold capillaroscopy
NVC: nailfold videocapillaroscopy
RP: Raynaud’s phenomenon
SLE: systemic lupus erythematosus
SSc: systemic sclerosis
UCTD: undifferentiated connective tissue disease
